# Overview and Methodology of the National HIV Behavioral Surveillance Among Transgender Women — Seven Urban Areas, United States, 2019–2020

**DOI:** 10.15585/mmwr.su7301a1

**Published:** 2024-01-25

**Authors:** Dafna Kanny, Kathryn Lee, Evelyn Olansky, Taylor Robbins, Lindsay Trujillo, Teresa Finlayson, Elana Morris, Christine Agnew-Brune, Susan Cha, Johanna Chapin-Bardales, Cyprian Wejnert, National HIV Behavioral Surveillance Among Transgender Women Study Group, National HIV Behavioral Surveillance Among Transgender Women Study Group, Narquis Barak, Kathleen A. Brady, Sarah Braunstein, Jasmine Davis, Sara Glick, Andrea Harrington, Jasmine Lopez, Yingbo Ma, Aleks Martin, Genetha Mustaafaa, Tanner Nassau, Gia Olaes, Jennifer Reuer, Alexis Rivera, William T. Robinson, Ekow Kwa Sey, Sofia Sicro, Brittany Taylor, Dillon Trujillo, Erin Wilson, Pascale Wortley

**Affiliations:** ^1^Behavioral and Clinical Surveillance Branch, Division of HIV Prevention, National Center for HIV, Viral Hepatitis, STD, and TB Prevention, CDC, Atlanta, Georgia; ^2^ICF, Fairfax, Virginia; ^3^Social & Scientific Systems, Inc., Atlanta, Georgia; ^4^Oak Ridge Institute for Science and Education, Oak Ridge, Tennessee; CrescentCare; Philadelphia Department of Public Health; New York City Department of Health and Mental Hygiene; CrescentCare; University of Washington, School of Medicine, Division of Allergy; and Infectious Diseases; Public Health – Seattle & King County, HIV/STD Program; Philadelphia Department of Public Health; New York City Department of Health and Mental Hygiene; Los Angeles County Department of Public Health; Public Health – Seattle & King County, HIV/STD Program; Georgia Department of Public Health; Philadelphia Department of Public Health; Los Angeles County Department of Public Health; Washington State Department of Health; New York City Department of Health and Mental Hygiene; Louisiana State University Health Science Center in New Orleans – School of Public Health; Louisiana Office of Public Health STD/HIV/Hepatitis Program; Los Angeles County Department of Public Health; San Francisco Department of Public Health; Georgia Department of Public Health; San Francisco Department of Public Health; San Francisco Department of Public Health; Georgia Department of Public Health.

## Abstract

Transgender women, especially transgender women of color, are disproportionately affected by HIV. However, no surveillance system collects data on HIV risk factors among this population. To address this gap, CDC developed a surveillance system entitled National HIV Behavioral Surveillance Among Transgender Women (NHBS-Trans) to assess behavioral and contextual data through systematic biobehavioral surveillance to monitor behavioral risk factors, prevention usage, and HIV prevalence among transgender women. NHBS-Trans used respondent-driven sampling in seven urban areas in the United States. Trained interviewers used a standardized, anonymous questionnaire to collect information on HIV-related behavioral risk factors, HIV testing, and use of prevention services. Each of the seven participating project areas recruited approximately 200 eligible transgender women and offered anonymous HIV testing. Overall, in the seven project areas, 1,757 participants completed the eligibility screener for NHBS-Trans during 2019–2020; of these, 6.6% were seeds (i.e., a limited number of initial participants who were chosen by referrals from persons and community-based organizations who knew or were part of the local population of transgender women). A total of 1,637 (93.2%) participants were eligible, consented, and completed the interview. Of these, 1,624 (99.2%) agreed to HIV testing. Of the total 1,637 participants, 29 participants did not report identity of woman or transgender woman, resulting in a final sample of 1,608 transgender women. NHBS-Trans project area staff members (n = 14) reported that the survey was timely and addressed a critical need for HIV surveillance in a population that is often overlooked. The *MMWR* supplement includes this overview report on NHBS-Trans, which describes the methods (history, participant eligibility criteria, questionnaire, data collection, and HIV testing) as well as evaluation of project implementation and the performance of the questionnaire content, specifically the acceptability for transgender women. The other NHBS-Trans reports in the supplement include information on pre-exposure prophylaxis use, psychosocial syndemic conditions and condomless anal intercourse, nonprescription hormone use, homelessness, discrimination and the association between employment discrimination and health care access and use, and social support and the association between certain types of violence and harassment (gender-based verbal and physical abuse or harassment, physical intimate partner abuse or harassment, and sexual violence) and suicidal ideation. NHBS-Trans provides important data related to the goals of the Ending the HIV Epidemic in the U.S. initiative. Findings from NHBS-Trans can help guide community leaders, clinicians, and public health officials in improving access to and use of HIV prevention and treatment services by transgender women.

## Introduction

CDC’s National HIV Behavioral Surveillance (NHBS) is a comprehensive system for biobehavioral surveillance (i.e., a surveillance activity that collects behavioral information through surveys and also collects biologic specimens for laboratory testing on disease status or biologic outcomes related to medication) that focuses on three core populations disproportionately affected by HIV: men who have sex with men, persons who inject drugs, and heterosexually active men and women at increased risk for HIV infection (*1*). Because HIV is disproportionally distributed among persons with low income, NHBS uses low income as a proxy for increased risk for acquiring HIV infection through heterosexual sex (*1*). However, CDC does not have a surveillance system that specifically focuses on HIV risk factors among transgender women, a group that is disproportionately affected by HIV, especially transgender women of color (*2*). Evidence suggests that in relation to their population size, transgender women are among the groups most affected by HIV in the United States (*2**,**3*). In 2017, CDC established the National HIV Behavioral Surveillance Among Transgender Women (NHBS-Trans) system. The goal was to conduct HIV-related biobehavioral surveillance to monitor behavioral risk factors, HIV testing behaviors, receipt of prevention services, use of prevention strategies, and HIV prevalence. The focus population was transgender women, defined as persons assigned male sex at birth but who identify as women or transgender women. Funding was awarded through the U.S. Department of Health and Human Services Secretary’s Minority HIV/AIDS Fund, formerly the Secretary’s Minority AIDS Initiative Fund, to focus on the recruitment of Black or African American and Hispanic or Latina transgender women (https://www.hiv.gov/federal-response/smaif/smaif-in-action). Two additional goals were to develop best practices for conducting biobehavioral surveillance with this population and to provide a platform for the funded health departments to work on community engagement.

CDC funded seven state and local health departments (hereafter referred to as project areas) to conduct NHBS-Trans in geographically diverse urban areas in the United States: Atlanta, Georgia (Georgia Department of Public Health), Los Angeles, California (Los Angeles County Department of Public Health); New Orleans, Louisiana (Louisiana Department of Health and Hospitals); New York City, New York (New York City Department of Health and Mental Hygiene); Philadelphia, Pennsylvania (Philadelphia Department of Public Health); San Francisco, California (San Francisco Department of Public Health); and Seattle, Washington (Washington State Department of Health).

This overview report describes NHBS-Trans 2019–2020 data and the system methods (history, participant eligibility criteria, questionnaire, data collection, and HIV testing) as well as evaluation of project implementation and the performance of the questionnaire content, specifically the acceptability for transgender women. The *MMWR* supplement also includes reports on pre-exposure prophylaxis use, psychosocial syndemic conditions and condomless anal intercourse, nonprescription hormone use, homelessness, discrimination and the association between employment discrimination and health care access and use, and social support and the association between certain types of violence and harassment (gender-based verbal and physical abuse or harassment, physical intimate partner abuse or harassment, and sexual violence) and suicidal ideation. Findings from NHBS-Trans can help guide community leaders, clinicians, and public health officials to improve access to and use of HIV prevention and treatment services by transgender women.

## Methods

### Overview

NHBS-Trans is an HIV-related biobehavioral surveillance system to monitor behavioral risk factors, prevention use, and HIV prevalence among transgender women. During 2019–2020, CDC conducted NHBS-Trans using respondent-driven sampling, a methodology similar to snowball sampling that is often used to sample hard-to-reach populations (*4*). This method relies on multiple waves of peer-to-peer recruitment to achieve the desired sample size. Applicable local institutional review boards in each participating project area approved NHBS-Trans activities. NHBS-Trans activities were described in the model surveillance protocol (https://www.cdc.gov/hiv/pdf/statistics/systems/nhbs/NHBS-Trans_Protocol.pdf). The final NHBS-Trans sample included 1,608 transgender women in seven urban areas in the United States (Atlanta, Georgia; Los Angeles, California; New Orleans, Louisiana; New York, New York; Philadelphia, Pennsylvania; San Francisco, California; and Seattle, Washington) recruited using respondent-driven sampling. This activity was reviewed by CDC, deemed not research, and was conducted consistent with applicable Federal law and CDC policy.^*^

### Formative Assessment

NHBS-Trans implementation started with 18 months (January 2018–June 2019) of formative assessment (https://www.cdc.gov/hiv/pdf/statistics/systems/nhbs/NHBS-Trans_Formative_Assessment_Manual.pdf). Formative assessment methods included a review of existing data, reports, and publications; qualitative interviews with key partner organizations, including service providers and community key informants; and focus groups (*5*). Project areas often used formative assessments to answer key implementation questions (e.g., the appropriate incentive for participation and safe, conveniently located field site locations for data collection). Project areas also used the formative assessment period to build community support for NHBS-Trans. Project areas assembled local community advisory boards (CABs), and project area staff members included transgender and gender nonconforming persons.

### Eligibility Criteria

NHBS-Trans included the following general eligibility criteria: aged ≥18 years, residence in a participating urban area, no previous participation in NHBS-Trans during the current data collection, ability to complete the interview in English or Spanish, and ability to provide informed consent. Additional eligibility criteria included reporting specific combinations of responses to sex listed at birth and gender identity questions (i.e., listed male at birth and gender identity including woman, transgender woman, or a gender not listed here or listed intersex at birth and a gender identity of transgender woman) (Box).

BOX **Criteria used to identify a transgender woman — National HIV Behavioral Surveillance Among Transgender Women, seven urban areas, United States, 2019–2020**

**Table:** 

**Sex listed at birth **	**Gender identity**				
	**Woman**	**Man**	**Transgender woman**	**Transgender man**	**A gender not listed here**
Male	Eligible	Not eligible	Eligible	Not eligible	Eligible
Female	Not eligible	Not eligible	Not eligible	Not eligible	Not eligible
Intersex	Not eligible	Not eligible	Eligible	Not eligible	Not eligible

### Questionnaire

The NHBS-Trans questionnaire was developed with the following guiding principles: focus on domains relevant to the lives of transgender women, preserve key NHBS indicators for comparability with other populations, and ensure questionnaire items are respectful and appropriate for transgender women. During August 2015–January 2016, CDC conducted a literature review to identify survey tools that have been used in studies that included transgender persons. As a starting point for the literature review, a draft of a systematic review table and the search strategies used to identify the articles included in the evidence table were provided (*6*). CDC replicated these search strategies to identify any new articles that had been published since the original search was performed (i.e., January 1, 2008–November 30, 2015) or articles describing a study of transgender persons that might contain a survey instrument but might not have met the inclusion criteria used in that systematic review. CDC identified 170 relevant articles including all 116 of the articles listed in the evidence table and 54 articles that were not included in the evidence table. Full-length copies of the articles identified by CDC searches were obtained and examined to identify all survey instruments that were used in the study described in the systematic review. CDC obtained contact information for 82 unique corresponding authors and retrieved 24 survey instruments. To this group of surveys obtained from the literature review, CDC added three surveys obtained from researchers who were conducting transgender studies but had not yet published their findings. All survey items were entered into a Microsoft Access database to assist in review of the survey items. This database allowed organizing and searching 4,256 survey items by domain, construct, and source.

CDC assembled a CAB to provide consultation on questionnaire development. The CAB included nine members: eight were transgender women and one was a cisgender woman with extensive research and clinical experience working with transgender women. The majority (n = 5) of CAB members were either Asian, Black or African American, or Hispanic or Latina (Hispanic), although more members were White than any other race and ethnicity. (Persons of Hispanic origin might be of any race but are categorized as Hispanic; all racial groups are non-Hispanic.) CAB members were recruited from each of the four major regions of the continental United States (Midwest, Northeast, South, and West) to ensure geographic representation.

Many questionnaire items were selected or adapted from the standardized set of questions used to collect information among the core NHBS populations. In addition, new questionnaire sections (e.g., gender identity and medical gender affirmation) were added to tailor the questionnaire for use among transgender women. The order of questions was designed to minimize the cumulative emotional toll of potentially distressing questions about stigmatized behavior, experiences of discrimination, assault, and suicidality. Certain measures of sociodemographic characteristics and social determinants of health among transgender women are common to all topics of reports included in the *MMWR* supplement (Table 1).

**TABLE 1:** Variables, questions, analytic coding, and measures — National HIV Behavioral Surveillance Among Transgender Women, seven urban areas,* United States, 2019–2020

Variable	Question	Analytic coding	Measure
Age	What is your date of birth?	(MM/YYYY)	Age group
Race	Which racial group or groups do you consider yourself to be in? You may choose more than one option.	American Indian or Alaska Native, Asian, Black or African American, Native Hawaiian or other Pacific Islander, or White	Race and ethnicity^†^
Ethnicity	Do you consider yourself to be of Hispanic, Latina, or Spanish origin?	Yes or no	
Education	What is the highest level of education you completed?	Never attended school; grades 1–8; grades 9–11; grade 12 or GED; some college, associate degree, or technical degree; bachelor’s degree; or any postgraduate studies	Education level
Household income, USD	What was your household income last year from all sources before taxes?	$0–$416 (M) or $0–$4,999 (Y), $417–$833 (M) or $5,000–$9,999 (Y), $834–$1,041 (M) or $10,000–$12,499 (Y), $1,042–$1,249 (M) or $12,500–$14,999 (Y), $1,250–$1,666 (M) or $15,000–$19,999 (Y), $1,667–$2,083 (M) or $20,000–$24,999 (Y), $2,084–$2,499 (M) or $25,000–$29,999 (Y), $2,500–$2,916 (M) or $30,000–$34,999 (Y), $2,917–$3,333 (M) or $35,000–$39,999 (Y), $3,334–$4,166 (M) or $40,000–$49,999 (Y), $4,167–$4,999 (M) or $50,000–$59,999 (Y), $5,000–$6,249 (M) or $60,000–$74,999 (Y), or ≥$6,250 (M) or ≥$75,000 (Y)	2019 poverty level^§^
	Including yourself, how many people depended on this income?	No. of dependents	
Disability	Are you deaf or do you have serious difficulty hearing? Are you blind or have serious difficulty seeing, even when wearing glasses? Because of a physical, mental, or emotional condition, do you have serious difficulty concentrating, remembering, or making decisions? Do you have serious difficulty walking or climbing stairs? Do you have difficulty dressing or bathing? Because of a physical, mental, or emotional condition, do you have difficulty doing errands alone, such as visiting a doctor’s office or shopping?	Yes or no	Disability status^¶^
Health care access	Do you currently have health insurance or health care coverage?	Yes or no	Health insurance status
	What kind of health insurance or coverage do you currently have?	A private health plan — through an employer or purchased directly Medicaid — for people with low incomes, Medicare — for the elderly and people with disabilities, some other government plan, TRICARE/CHAMPUS, Veterans Administration coverage, or some other health insurance	Type of health insurance
	In the past 12 months, that is, since [fill with interview month, formatted as text] of last year, have you seen a doctor, nurse, or other health care provider?	Yes or no	Recent health care use
	Do you have a health care provider with whom you feel comfortable discussing gender-related health issues?	Yes or no	Transgender-specific health care access
	Does your current health insurance cover hormones for gender transition or affirmation?	Yes or no	Transgender-specific health insurance coverage
Homelessness	In the past 12 months, have you been homeless at any time? By homeless, I mean you were living on the street, in a shelter, in a single room occupancy hotel (SRO), or in a car.	Yes or no	Experienced homelessness
Incarceration	During the past 12 months, have you been held in a detention center, jail, or prison for >24 hours?	Yes or no	Incarceration
Exchange sex	In the past 12 months, have you received money or drugs in exchange for sex?	Yes or no	Exchange sex
Food insecurity	In the past 12 months, did you ever cut the size of your meals or skip meals because there wasn’t enough money for food? In the past 12 months, did you ever not eat for a whole day because there wasn’t enough money for food?	Yes or no	Food insecurity
Abuse and harassment	In the past 12 months, have you been verbally abused or harassed because of your gender identity or presentation?	Yes or no	Verbal abuse (gender-based violence)
	In the past 12 months, have you been physically abused or harassed because of your gender identity or presentation?	Yes or no	Physical abuse (gender-based violence)
	In the past 12 months, have you been physically abused or harassed by a sexual partner?	Yes or no	Physical intimate partner violence
	In the past 12 months, have you been forced to have sex when you did not want to? By forced, I mean physically forced or verbally threatened. By sex, I mean any sexual contact.	Yes or no	Forced sex (sexual violence)

**Abbreviations:** GED = General Educational Development; M = monthly; Y = yearly; USD = U.S. dollars.

* Atlanta, GA; Los Angeles, CA; New Orleans, LA; New York City, NY; Philadelphia, PA; San Francisco, CA; and Seattle, WA.

^† ^Persons of Hispanic or Latina (Hispanic) origin might be of any race but are categorized as Hispanic; all racial groups are non-Hispanic.

^§^ 2019 Federal poverty level thresholds were calculated on the basis of U.S. Department of Health and Human Services Federal poverty level guidelines (https://aspe.hhs.gov/topics/poverty-economic-mobility/poverty-guidelines/prior-hhs-poverty-guidelines-federal-register-references/2019-poverty-guidelines).

^¶^ To assess difficulty in six basic domains of functioning (hearing, vision, cognition, walking, self-care, and independent living), based on U.S. Department of Health and Human Services disability data standard (https://aspe.hhs.gov/reports/hhs-implementation-guidance-data-collection-standards-race-ethnicity-sex-primary-language-disability-0).

### Data Collection

During June 2019–February 2020, project areas collected biobehavioral and contextual data implementing recruitment and operational procedures (https://www.cdc.gov/hiv/pdf/statistics/systems/nhbs/NHBS-Trans_Operations_Manual.pdf). Recruitment started with a limited number of initial participants (i.e., seeds) who were referred by community-based organizations and persons from the local population of transgender women. Initial recruits who completed the eligibility screener and were eligible were interviewed, and those who completed the interview were asked to recruit up to five transgender women whom they knew personally. Those persons, in turn, completed the interview and were asked to recruit others using a system of coded coupons. Participants whose sex listed at birth was male and their gender identity included a gender not listed here but did not include woman or transgender woman could not recruit others. The recruitment process continued until the sample size was reached or data collection ended. Project area staff members conducted recruitment and data collection activities at established field sites (e.g., health department or community-based organization’s offices) or in a mobile van parked in an established location at a field site.

Each of seven participating project areas planned to recruit approximately 200 eligible transgender women. Recruited and consented participants completed an interviewer-administered, standardized, in-person anonymous questionnaire using computer tablets. Key questionnaire components included demographics, sexual behaviors, alcohol use, injection and noninjection drug use, HIV testing experiences, health conditions, access to care and prevention activities, gender-affirming medical treatment, social support, experiences of abuse and harassment, and mental health, including suicidality. Each interview took an average of 40 minutes to complete (https://www.reginfo.gov/public/do/PRAViewICR?ref_nbr=201902-0920-007 andhttps://www.cdc.gov/hiv/pdf/statistics/systems/nhbs/NHBS-Trans_CRQ.pdf).

### HIV Testing

NHBS-Trans offered all participants anonymous, blood-based rapid HIV testing. Participants who did not self-report a previous HIV diagnosis and had a first rapid test that was reactive received a second orthogonal rapid test (i.e., rapid-rapid testing algorithm) to confirm infection. Nonlaboratory staff members in project areas conducted HIV rapid tests in field settings under Clinical Laboratory Improvement Amendments waivers (https://www.cms.gov/regulations-and-guidance/legislation/clia/downloads/howobtaincertificateofwaiver.pdf). Participants received their HIV test results after completing the interview and were referred to treatment and other health and social services as needed. Participants who self-reported a previous HIV diagnosis, had at least one reactive HIV rapid test, or both, and consented to specimen storage, provided dried blood spot specimens for future laboratory testing (e.g., HIV viral load testing at CDC). Participants received incentive payments (e.g., a gift card) for the interview and HIV testing in person. Participant compensation for incomplete surveys could be offered in accordance with local policies. Incentives were given to those interviewed and tested for HIV (approximately $25 for each). Additional rewards (approximately $10) were paid to those who successfully recruited others. Local project areas determined the amount and type of incentives deemed appropriate for the local populations being interviewed and tested.

### Qualitative Evaluation

During data collection, CDC conducted a surveillance system evaluation using semistructured qualitative interviews (i.e., interviews with a script of open-ended questions) with 14 key project area staff members across project areas to assess their experiences with staff member support, NHBS-Trans project development and implementation, and community engagement (Rushmore J, CDC, unpublished data, 2019). The interview guide is available (Supplementary Interview Guide, https://stacks.cdc.gov/view/cdc/137444). After data collection concluded, CDC conducted a questionnaire evaluation among project area interviewers to assess the general performance of questions, with a focus on acceptability of questionnaire items for transgender women.

## Results

Overall in the seven project areas, 1,757 participants completed the eligibility screener for NHBS-Trans; of these, 6.6% were seeds (Table 2). Throughout data collection as part of the recruitment process, 5,642 coupons were distributed to participants to recruit their peers. A total of 1,637 (93.2%) participants were eligible, consented, and completed the interview. Of these, 1,624 (99.2%) agreed to HIV testing. Of the total 1,637 participants, 29 participants did not report identity of woman or transgender woman, resulting in a final sample of 1,608 transgender women.

**TABLE 2:** Number and percentage of screened participants, seeds,* distributed coupons, records, and HIV testing, by project area — National HIV Behavioral Surveillance Among Transgender Women, seven urban areas,^†^ United States, 2019–2020

Project area	No. of screened participants	Participants who were seeds No. (%)	Distributed coupons No. (%)	Records^§^ No. (%)	Agreed to HIV testing^¶^ No. (%)	No. of transgender women in final sample
Atlanta, GA	164	15 (9.2)	650	136 (82.9)	134 (98.5)	132
Los Angeles, CA	523	6 (1.2)	1,464	505 (96.6)	504 (99.8)	504
New Orleans, LA	192	20 (10.4)	708	177 (92.2)	174 (98.3)	165
New York City, NY	303	10 (3.3)	930	281 (92.7)	278 (98.9)	279
Philadelphia, PA	223	12 (5.4)	597	220 (98.7)	218 (99.1)	220
San Francisco, CA	214	26 (12.2)	780	201 (93.9)	201 (100.0)	198
Seattle, WA	138	27 (19.6)	513	117 (84.8)	115 (98.3)	110
**Total**	**1,757**	**116 (6.6)**	**5,642**	**1,637 (93.2)**	**1,624 ** **(99.2)**	**1,608**

* A limited number of initial participants who were chosen by referrals from persons and community-based organizations who knew or were part of the local population of transgender women.

^†^ Atlanta, GA; Los Angeles, CA; New Orleans, LA; New York City, NY; Philadelphia, PA; San Francisco, CA; and Seattle, WA.

^§^ Total number of records includes the number of records for participants who were eligible, consented to the survey, completed the interview, and for whom the interviewer was confident or somewhat confident in the responses to the interview questions.

^¶^ Among total number of records.

All NHBS-Trans project area staff members who participated in the qualitative evaluation reported that the survey was timely and addressed a critical need for HIV surveillance in a population that is often overlooked. Although certain project areas reported recruiting a sample diverse in age, race and ethnicity, and socioeconomic status, others experienced challenges with recruiting key subgroups (e.g., younger women and Hispanic participants). In addition, certain project area staff members indicated concerns with acceptability of interview questions among transgender women. Differences in opinions to expand the recruiter eligibility criteria allowing gender nonconforming participants to recruit from their networks were observed by geographic region (e.g., East Coast versus West Coast). Staff members emphasized the importance of community support and relationships for ensuring the success of the surveillance system.

Interviewers in seven project areas collectively provided feedback on 117 questionnaire items. The majority (51%) of the feedback concerned the sections that were added specifically for transgender women populations (gender identity [21%] and medical gender affirmation [9%]) and the sex behavior section (21%), which was adapted from the core NHBS questionnaire. Major themes identified through interviewer feedback included interview flow, research mistrust, clarity of certain questions about pre-exposure prophylaxis adherence and homelessness, Spanish translation, transgender cultural competency, and the need for improvements in a trauma-informed approach to particularly sensitive questions (e.g., available referrals for crisis counseling for participants, managerial support for staff members experiencing secondary trauma, and disclaimers and introductions that explain the sensitive nature of the questions) (https://store.samhsa.gov/sites/default/files/sma14-4884.pdf). This feedback will be used to guide future iterations of the NHBS-Trans questionnaire.

## Discussion

Approximately 1,600 eligible transgender women from seven project areas participated in the first NHBS-Trans during 2019–2020. Data from NHBS-Trans have reaffirmed that transgender women need to be a priority population in preventing HIV infection (*7*). The disproportionate effect of HIV infection among transgender women is the result of a complex layering of syndemics, and more remains to be understood (*8*). NHBS-Trans highlights the social and economic factors that are contributing to this disparity.

NHBS-Trans data have been used to provide behavioral and community context for trends in HIV infection diagnoses reported to CDC’s National HIV Surveillance System (https://www.cdc.gov/hiv/statistics/surveillance/index.html). NHBS-Trans data also have described a population disproportionately affected by HIV and, thus, have provided indications of the leading edge of the epidemic. CDC, along with project areas, has been disseminating these data. Dissemination products have included the HIV Surveillance Special Report (*7*) and infographics (https://www.cdc.gov/hiv/pdf/library/reports/surveillance/cdc-hiv-surveillance-special-report-number-27-infographic.pdf), presentations (https://www.cdc.gov/hiv/pdf/statistics/systems/nhbs/cdc-hiv-nhbs-transgender-women-surveillance-report-2019-2020.pdf), *MMWR* (*9*), and peer-reviewed journals (*10**–**13*).

Participants in NHBS-Trans were offered HIV testing and referral to care, if test results were positive. Providing HIV testing and the resources to connect to care enabled NHBS-Trans participants to know their status, seek treatment, or engage in future prevention strategies. Further, biologic information on HIV status via rapid-rapid testing algorithms can identify gaps in HIV screening and prevention efforts for transgender women.

A strong connection to the transgender communities in each project area was crucial for recruitment of participants and successful data collection. Project areas spent a year identifying and hiring staff members and learning about and engaging with their communities. They assembled local CABs and strengthened relations between transgender communities and their health departments. Buy-in from partner organizations was critical to the initiation and success of the project.

In every project area, transgender and gender nonconforming persons often comprised most front-line staff members and in many cases were in managerial roles. Including transgender and gender nonconforming persons as project area staff members was critical to connecting with local communities and, consequently, the success of NHBS-Trans. Further, in many cities, these staff members remained in permanent health department positions after the project ended and continued to serve as community liaisons and representatives of transgender women in their cities.

Throughout the preparation for NHBS-Trans, project areas explored various methods for sharing data, understanding the importance of returning findings back to the community. Efforts are ongoing to engage the community (i.e., local data analysis presentations tailored to the priorities of local transgender women partner organizations [https://www.youtube.com/watch?v=rPEBTXUXheA] and shared information with community members), ensuring that persons who provided these data are positioned to receive and benefit from it (https://www.nyc.gov/assets/doh/downloads/pdf/dires/hiv-transgender-women-factsheet.pdf).

## Limitations

The findings in the supplement are subject to at least five limitations. First, NHBS-Trans data are not nationally representative and might not be generalizable to all U.S. urban areas, nonurban areas, or all transgender women. However, the hidden and hard-to-reach nature of this population prevents collection of nationally representative samples. Second, respondent-driven sampling has certain sources of bias. Groups that are more insular (i.e., more likely to recruit only within their own group) are more likely to be overrepresented (if recruitment chains become trapped inside the group) or underrepresented (if recruitment chains cannot access the group) in the sample than less insular groups (*14*). Groups with larger networks might be overrepresented in the sample because more recruitment paths lead to their members. Certain groups might be less willing or able to participate in the survey and would be underrepresented in the sample. This bias can be assessed and compensated for in multiple ways. Certain potential sources of bias were identified and addressed by NHBS-Trans project area staff members. For instance, project area staff members were encouraged to ensure that their initial peer recruits, or seeds, were diverse by race and ethnicity, age, geographic location, and other important factors that would have the effect of increasing the insularity of recruitment and of homophily (i.e., groups that recruit only within their own group). Project areas also implemented lessons learned during formative assessment to mitigate potential participation bias. For example, information from formative assessment was used to optimize location and setup of field sites so that all population members had safe, convenient access to participants (*15**,**16*). Third, biases in enrollment and agreement to HIV testing might result in over- or underestimation of HIV infection prevalence or incidence. If those who agree to be tested differ from those who decline, in terms of age, race and ethnicity, or sex, findings might be less generalizable. Fourth, because NHBS-Trans was a one-time cross-sectional survey, causality or directionality of the findings cannot be determined. Finally, the data are self-reported and are subject to recall and social desirability biases.

## Conclusion

NHBS-Trans collected for the first time behavioral and contextual data through systematic biobehavioral surveillance of transgender women from seven participating project areas during 2019–2020. A strong connection to the transgender communities in each project area was crucial for recruitment of participants and successful data collection. NHBS-Trans findings highlighted in the *MMWR* supplement can help guide community leaders, clinicians, and public health officials’ efforts in improving access to and use of HIV prevention and treatment services by transgender women.
